# The Evolution of Chimeric Antigen Receptor T-Cell Therapy in Children, Adolescents and Young Adults with Acute Lymphoblastic Leukemia

**DOI:** 10.3390/biomedicines10092286

**Published:** 2022-09-14

**Authors:** Dristhi Ragoonanan, Irtiza N. Sheikh, Sumit Gupta, Sajad J. Khazal, Priti Tewari, Demetrios Petropoulos, Shulin Li, Kris M. Mahadeo

**Affiliations:** 1 Department of Pediatrics, Stem Cell Transplantation and Cellular Therapy, The University of Texas MD Anderson Cancer Center, Houston, TX 77030, USA; 2 Department of Pediatrics Research, The University of Texas MD Anderson Cancer Center, Houston, TX 77030, USA

**Keywords:** acute lymphoblastic leukemia, ALL, chimeric antigen receptor, CART, CD19, CD22, Bispecific CART

## Abstract

Chimeric antigen receptor T-cell (CAR T) therapy is a revolutionary treatment for pediatric, adolescent and young adult patients (AYA) with relapsed/refractory B-cell acute lymphoblastic leukemia. While the landscape of immunotherapy continues to rapidly evolve, widespread use of CAR T therapy is limited and many questions remain regarding the durability of CAR T therapy, methods to avoid CAR T therapy resistance and the role of consolidative stem cell transplant. Modified strategies to develop effective and persistent CAR T cells at lower costs and decreased toxicities are warranted. In this review we present current indications, limitations and future directions of CAR T therapy for ALL in the pediatric and AYA population.

## 1. Introduction

Acute lymphoblastic leukemia (ALL) is the most common pediatric malignancy with 60% of all cases diagnosed before the age of 20. While the overall survival for pediatric patients with ALL has dramatically improved over the past four decades to 80–90%, 15–20% of patients eventually relapse [1]. The treatment for pediatric patients with relapsed/refractory (R/R) ALL remains challenging and their prognosis is poor with conventional salvage chemotherapy. Novel therapeutic strategies are urgently needed. As the immune landscape continues to evolve, chimeric antigen receptor T-cell (CAR T-cell/CAR T) therapy has emerged as a promising treatment option for patients with R/R ALL, with impressive remission rates of 70–90% [2,3,4]. In 2017, the first CAR T-cell therapy, Tisagenlecleucel, was approved by the United States Food and Drug Administration (FDA) for the treatment of patients ≤25 years of age with R/R CD19+ B-cell acute lymphoblastic leukemia (B-ALL) who have experienced a second or greater relapse [5]. Due to the improvements in treatment of R/R disease using CAR T-cell therapy, it is important to explore the role that CAR T-cell therapy may have as a frontline treatment option, as it would allow targeted treatment with possibly reduced morbidity and improved quality of life.

Despite its initial success in patients with R/R ALL, however, longer follow-up of patients post-CAR T-cell therapy have revealed clinically significant limitations. These include manufacturing time, suboptimal CAR T-cell function, tumor cell resistance, immunotoxicities and failure to maintain durable disease remission. This review will focus on current available CAR T-cell therapies for B-ALL, their limitations, future directions and expanding indications for this therapy.

## 2. Methods

We searched PubMed, Embase and Clinicaltrials.gov for articles and reviews published between 1 June 2016 and 26 October 2021, as well as ongoing clinical trials. Older references were also used when appropriate. We used the search terms “acute lymphoblastic leukemia”, “B-ALL”, “T-ALL”, “CD5”, “CD7”, “CD19”, “CD22”, “Bispecific CART”, “chimeric antigen receptor”, “CART”. We also searched abstracts from relevant conferences including “American Society of Hematology”, “American Society for Transplantation and Cellular Therapy” and “European Society for Blood and Marrow Transplantation”. References from articles and abstracts reviewed were also cross-referenced.

## 3. Generations of Chimeric Antigen Receptors

Chimeric antigen receptors (CARs) are synthetic hybrid receptors that are composed of an extracellular, antigen recognition-binding domain usually derived from the single-chain variable fragment(scFv) of an immunoglobulin, a hinge region, a transmembrane domain and the CD3ζ intracellular domain of the T-cell receptor [6]. Multiple generations and modifications of these CAR T cells have been genetically created ex vivo. It should be noted that while each successive CAR T-cell generation has the addition of certain transmembrane or interleukin receptor (ILR) domains, the simple addition of each of these does not necessarily lead to a more effective CAR T-cell construct. An important consideration is balancing the activation of CAR T cells through transmembrane small molecule activation in order to direct cytotoxic activity and limit toxicities such as cytokine release syndrome (CRS) and severe neurotoxicity [7,8].

First generation CAR T cells initially contained only the CD3-ζ activation domain and resulted in poor in vivo proliferation and persistence as they failed to sufficiently activate the CAR T cells [9].

Subsequent generations of CAR constructs added one (2nd generation) or two (3rd generation) costimulatory molecules to the intracellular domain to further boost the activation signaling of T cells, the most common being CD28, 4-1BB (CD137) and/or OX40. Fourth generation CARs combine additional factors such as cytokine receptors to further stimulate antitumor activity and downregulate CAR T-cell exhaustion markers such as T- cell immunoglobulin domain and mucin domain-containing protein 3 (TIM-3) and lymphocyte activation gene 3 protein (LAG-3) (Table 1) [3,10,11,12]. The development of 5th generation CAR T cells is also underway and focuses on the development of inserting interleukin-2 (IL-2) receptor chains along with co-stimulatory domains in order to facilitate cytokine signaling through the activation of the JAK-STAT pathway. This serves to not only activate the CAR T cell through an antigen-dependent pathway, but also leads to persistent effector function against CD19 due to activation of multiple costimulatory domains [13].

While the majority of trials have been with autologous CAR T-cell products, allogeneic off-the-shelf products are also being explored as “ready to use” products, allowing greater accessibility of CAR T-cell products [14].

## 4. Outcomes of CAR T-Cell Therapy

Multiple CAR T-cell therapy constructs with varying costimulatory domains and target antigens have been trialed with varied responses reported amongst clinical trials (Table 2). Below, we review the differences in effect due to the varying costimulatory domains as well as the antigens directed in CAR T-cell therapy and highlight areas in which improvements in the manufacturing of CAR T cells may be indicated. It should be noted that the responses in Table 2 may be an overestimation. Not all patients with R/R disease in each study received CAR T cells due to possible manufacturing failures and/or rapidly advancing disease status and/or death prior to the opportunity for infusion.

In the landmark phase II ELIANA trial of 75 pediatric and young adult patients with R/R ALL receiving tisagenlecleucel (anti-CD19 CAR construct), 81% of patients achieved complete remission (CR), all with no minimal residual disease at 3 months. Overall survival (OS) was 90% and 76%, and event-free survival (EFS) was 73% and 50%, at 6 and 12 months, respectively [4]. However, the median EFS was not reached. Thus, strategies to optimize durable remissions either through development of new CAR T constructs and/or further consolidative therapies may be indicated [33].

As an alternative option to those that failed anti-CD19 therapy, Fry et al. [18] reported the use of anti-CD22 CAR with a 4-1BB costimulatory domain in 21 children and adults (median age 19 years old) in a phase I trial. Fifteen patients received prior anti-CD19 CAR T therapy and an additional two were previously treated with CD19-directed immunotherapy. Twelve patients (57%) achieved complete remission, nine of whom were negative for minimal residual disease (MRD). Eight patients subsequently relapsed and median remission duration was 6 months (1.5–12 months). Another phase 1 study (NCT04036019) indicated that in patients with R/R non-Hodgkin lymphoma who failed anti-CD19 CAR T therapy, the use of an anti-CD20 CAR construct was safe in that 86% of patients experienced only grade 1 or 2 CRS with no severe neurotoxicity [34]. While anti-CD20- or CD22- directed CAR T-cell therapy may have a role in the treatment of pediatric patients with R/R ALL who relapse following anti-CD19 CAR T-cell therapy, it is evident that improved CAR constructs may be needed in order to achieve durable remission. In this scenario, the use of a dual targeting CAR T-cell construct may be of utility. Indeed, Spiegel et al. [35] have demonstrated through their development of a bispecific CAR targeting CD19/CD22, in a phase 1 clinical trial (NCT03233854), that such a construct had utility in adult patients with R/R B cell malignancy. In this trial, 88% of patients with B-ALL demonstrated MRD- negative complete remission (CR) following treatment with a CD19/CD22-directed dual CAR T cell.

The effect of the costimulatory domain on CAR T-cell efficacy has varied based on reports. In a systematic review and meta-analysis of 11 studies comprised of 346 pediatric patients (aged <18 years), 81% of patients achieved CR with no significant difference between 4-1BB and CD28 co-stimulated CAR T constructs [36]. In another meta-analysis of 15 pediatric and AYA studies of 448 patients aged <30 years receiving CAR T therapy, the CR rate was 82% and MRD-negative CR rate was 78%. Subgroup analysis showed the incidence of MRD-negative CR varied by CAR T-cell construct with a rate of 69% in three studies that utilized the CD28z costimulatory domain, 77% in one study using a fourth-generation CAR T-cell product and 81% in 11 studies using a 4-1BB domain CD19-specific CAR T product. The cumulative incidence rates of relapse with CD19-CD28z and CD19–4-1BB were 0.28 (95% CI, 0.17–0.48; p = 0.61) and 0.36 (95% CI, 0.30–0.47; p = 0.28), respectively [37].

Another exciting development in the evolution of CAR T-cell therapy is the development of a universal CAR T (UCAR T) product where allogeneic CAR T cells are taken from healthy donors and modified through the use of transcription activator-like effector nucleases (TALENs). In this method, the development of a CAR T cell in which mRNA is used to knock out genes related to the production of receptor proteins associated with graft versus host disease (GVHD) is currently under investigation [38]. Preliminary data on the use of UCAR-T19 (allogeneic anti-CD19 scFv-41BB-CD3ζ construct) in 21 pediatric (n = 7) and adult (n = 14) patients reported 67% of patients achieved CR at day 28 with a median response duration of 4.1 months. Progression-free survival (PFS) and OS at 6 months was 27% and 55%, respectively. Cytokine release syndrome (CRS) and immune effector cell–associated neurotoxicity syndrome (ICANS) of any grade were reported in 91% and 38% of patients, respectively. No serious neurotoxicity was reported, and two patients developed grade 1 cutaneous graft vs. host disease (GVHD) [39]. In the BALLI-01 Phase 1 trial (NCT04150497), five adult patients at a median age of 24 years (22–52 years) with R/R B-ALL received UCAR-T22 (allogeneic anti-CD22/41BB construct). Two patients achieved CR with incomplete hematologic recovery (CRi) at day 28, one of which subsequently received inotuzumab ozogamicin followed by HSCT. Three patients had CRS (max grade 2) and there was no reported ICANS or GVHD [40,41]. No dose-limiting toxicities were reported and the study has since expanded its eligibility criteria and is now open to pediatric patients aged 15 years and older. The above studies offer a promising strategy for patients who may not be eligible for autologous CAR T therapy due to low and/or impaired lymphocyte counts/function.

As the experience with CAR T therapy continues to grow, the likelihood of durable remission post CAR T-cell therapy remains to be defined and requires discussion of modalities such as post-CAR T-cell therapy hematopoietic stem cell transplantation (HSCT). In the ELIANA trial, while 81% of patients achieved CR, this remission was not durable and relapse-free survival (RFS) decreased to 59% (95% CI, 41 to 73%) 12 months post-infusion. Eight of these patients (8/75, 13%) underwent HSCT and were all alive at the time of publication (four of whom were in remission) [4]. Talleur et al. [42] reported that 15 of 22 patients (68.2%), aged 1.8–23.6 years, who received either tisagenlecleucel (n = 10) or SJCAR(FMC63-based CD19-binding domain, 41bb construct) (n = 12), achieved clinical remission. Eighty six percent of these patients (n = 13/15) had MRD-negative disease. Of the patients who achieved CR, six patients proceeded to receive HSCT at a median of 67.5 days post-infusion and CR was maintained. Of the eight patients who did not receive HSCT, six relapsed at a median of 153 days (50–271 days) post-infusion. Lee et al. and Gardner et al. [10,43] also reported higher relapse rates in patients who were in CR post- CAR T-cell therapy without subsequent consolidative HSCT. Given the risk of relapse following CAR T-cell therapy, current data suggest that consolidative therapy in the form of HSCT may be an appropriate option as the above studies demonstrate positive CR rates in those that receive HSCT following CAR T-cell therapy. This is particularly important in patients with early loss of B-cell aplasia which indicates the loss of CAR T-cell effector function and higher risk of early relapse [44]. However, the use of post-CAR T-cell therapy HSCT remains an area of debate and future randomized trials are needed [45,46,47].

## 5. CAR T Therapy and Extramedullary Disease

**Figure 1:**
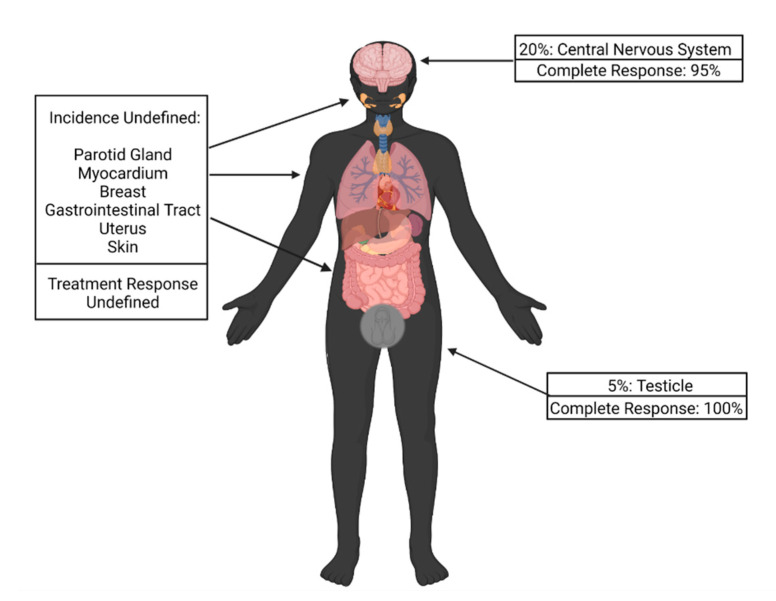
Incidence of extramedullary disease relapse by site and response to chimeric antigen receptor T-cell therapy.

Up to 20% of patients with relapsed ALL may develop extramedullary (EM) disease relapse. While the most common sites are the central nervous system (CNS) in 20% of cases and testes in 5% of cases, other sites reported include the parotid gland, gastrointestinal tract, breast, uterus and skin (Figure 1) [48,49,50].

Moreover, EM disease can occur in isolation or concurrently with bone marrow relapse, with the latter associated with poorer overall survival [21,51,52,53].

Treatment of EM disease conventionally includes systemic chemotherapy and radiation with varying outcomes. Consolidative HSCT has also been used; however, a multicenter trial showed an increase in treatment-related mortality (TRM), comparing patients who received matched sibling HSCT versus those who received systemic chemotherapy only for isolated CNS ALL relapse (22% vs. 9%, respectively) [54]. Additionally, radiation can have significant adverse effects. Testicular radiation, for example, can result in delayed puberty, need for hormone replacement, infertility and sexual dysfunction. As the number of childhood ALL survivors increases, novel therapeutic options are needed to decrease the possibility of long-term adverse effects [55].

CAR T therapy as a single agent has been reported to induce durable remission in patients with EM disease; however, experience is limited. In a multi-trial analysis of 182 patients, Newman et al. [56] reported a 95% CR rate among the 65 patients with R/R CNS B cell ALL at one month post-antiCD19 CAR T-cell infusion. The median age of patients enrolled was 10 years (1–29 years). Despite concerns in early trials for increased neurotoxicity in patients with CNS disease, neurotoxicity of any grade was similar amongst patients with CNS disease and those without (52% vs. 40%; 12% vs. 11% grade 3 or 4; p = 0.41). RFS was also similar between the two groups at 61% and 60%, respectively. Rubinstein et al. [57] reported similar findings of CR and low incidence of neurotoxicity in six patients with isolated CNS ALL relapse treated with tisagenlecleucel. Varying degrees of responses have also been reported with the use of CD19 and CD22 CAR T therapies for the treatment of non-CNS EM disease [18,50,58]. Of 10 pediatric patients with EM disease, Talekar et al. [51] reported RFS of 60% at a median follow up of 10 months (3–16 months). Chen et al. [52] reported CR of isolated testicular ALL relapse in seven pediatric patients. One patient subsequently had isolated BM relapse and the RFS for the other six patients was a median of 14 months (5–23 months). The overall 1 year EFS was 83.3% with no significant adverse events reported and a max CRS of grade 1 occurring in five patients. Leahy et al. [59] demonstrated no significant difference in CR, RFS and/or OS between pediatrics patients receiving anti-CD19 CAR T therapy with CNS R/R ALL, versus those without CNS disease. When comparing patients with CNS disease and those without, the CR was non-significant at 28 days (97% vs. 94%, respectively). Rates of severe neurotoxiciy and CRS also did not differ between the two groups. Moreover, in patients with isolated CNS disease, 2 year OS was statistically significant (91% vs. 71%, respectively) when compared to patients with bone marrow disease. In aggregate, these studies suggest that CAR T therapy is a promising strategy for patients with CNS relapse, with or without bone marrow disease, including patients with isolated CNS relapse [59].

While these small studies are promising, CAR T cells may not be able to successfully traffic to all sites of EM disease and additional targeted treatment may be required [60]. Additional studies show that the tumor microenvironment of these EM sites may differ and have increased regulatory T cells (Tregs) and myeloid-derived suppressor cells leading to greater inhibition of the CAR T cells and immunotherapy resistance [61]. Large-scale long-term prospective studies are needed to understand the optimal timing of CAR T therapy, the role of radiation in combination with CAR T therapy as it may potentially enhance the tumor microenvironment and enhance CAR T-cell function, and the long-term efficacy of CAR T therapy in patients with EM disease, including CNS disease, and associated toxicities.

## 6. CAR T Therapy Resistance and Durability

Despite achieving impressive CR rates in multiple clinical trials, 40–50% of patients eventually relapse post-CAR T therapy [4,62]. Antigen loss has emerged as a major mechanism of CAR T therapy resistance and disease relapse. Antigen loss has been reported to be due to a number of factors including upregulation of alternatively spliced CD19 transcripts, epitope masking, lineage switching and acquisition of myeloid markers and genetic mutations in CD19, resulting in the loss of the targeted surface antigen [63,64,65,66]. Grupp et al. [62] reported in a cohort of 50 patients who achieved remission with anti-CD19 CAR T-cell therapy, 40% of patients relapsed at a median follow-up of 10.6 months and loss of the CD19 antigen accounted for 65% of relapses, indicating that there is a need for alternate-antigen-directed CAR T-cell therapy in order to bypass the loss of a specific antigen such as CD19. Even in patients in which dual directed therapy may be used, antigen escape has been reported in both CD19- and CD 22-directed CAR T-cell therapy in pediatric ALL, further highlighting the need for novel strategies to improve tumor cell targeting. Diminished target density as opposed to complete antigen loss has also been reported as an escape mechanism [18]. Alternative target antigens or dual and triple targeted immunotherapies may be a strategy to overcome antigen loss and immunotherapy resistance to a single target CAR. Multiple groups have reported preliminary studies using a multitargeted approach; however, larger studies are needed to determine its efficacy and safety profile [67,68,69] (Table 3).

A related approach currently under investigation is the use of sequential single targeted CAR T-cell infusion products as reported by Liu et al. [75]. Here, sixteen patients with relapsed ALL post-allogeneic HSCT received sequential CAR T-cell infusions with anti-CD19 and anti-CD22 CAR T-cells. At a median follow-up of 6.5 months, the OS was 100% and disease-free survival (DFS) was reported at 81.3%. CRS occurred in 91.7% of patients receiving the first CAR T-cell infusion while the second infusion resulted in a maximum of grade I CRS. This low level of toxicity was attributed to the low leukemia burden. Twenty nine percent of patients, however, developed GVHD in which two patients were reported to have severe hepatic GVHD, resulting in one death. Liu et al. [] also report preliminary data in a clinical trial of pediatric patients with B-cell lymphoid malignancies, including non-Hodgkin lymphoma, in regard to the use of anti-CD19, anti-CD20, or anti-CD22 CAR T-cell therapy in a sequential manner. Their results indicate a significant overall response rate of 94% and CR of 71%, with a little more than half of patients experiencing CRS and more than 40% experiencing neurotoxicity. Overall, it appears that the use of sequential CAR T-cell infusion may have utility, especially due to positive initial response rates; however, further investigation is required to improve the safety profile to decrease the rate of CRS, neurotoxicity, and the incidence and prevention of GVHD.

T-cell exhaustion is another mechanism believed to contribute to poor CAR T-cell persistence. Checkpoint inhibitors may improve CAR T-cell persistence and function by inhibiting the PD-1: PD-L1 checkpoint axis, thereby decreasing T-cell exhaustion. Li et al. [76] reported the use of pembrolizumab (n = 13) or nivolumab (n = 1) in patients aged 4–17 years with relapsed B-ALL (n = 13) or B lymphoblastic lymphoma (n = 1)14 days or later after anti-CD19-directed CAR T infusion. Three of the six patients treated with anti-CD19 CAR T-cells in combination with a PD-1 inhibitor for early B-cell recovery, re-established B-cell aplasia for 5–15 months. Adverse effects associated with PD-1 inhibition were reported as: one case each of acute pancreatitis, hypothyroidism, arthralgias, urticaria, as well as four patients with grade 3–4 cytopenias and three patients with CRS symptoms within 2 days of starting pembrolizumab. No grade 5 AE or GVHD flare occurred. Interestingly, two patients discontinued pembrolizumab for delayed adverse effects and both relapsed/progressed with CD19+ disease a few weeks after discontinuation.

The CAR T construct itself can also play a role in CAR T-cell persistence, with 4-1BB CD28 costimulatory domains shown to reduce or augment exhaustion [77]. Several studies reported a prolonged persistence of anti-CD19 CAR T cells with a 4-1BB domain versus a CD28–based CAR construct [3,21]. The duration of CAR T-cell persistence has also varied amongst products with a median in vivo persistence of 168 days for 19-BBZ CAR T compared to 30 days with a 19–28z CAR T-cell construct [4]. Overall, these studies indicate that the domains used in the manufacturing of the CAR T-cell products play a role in decreasing the rate of T-cell exhaustion.

Pretreatment variables may also play a role in the efficacy of CAR T therapy. Pillai et al. [78] reported that patients who received prior treatment with blinatumomab (n = 16) prior to anti-CD19 CAR T therapy had a lower MRD-negative remission rate and higher rate of CD19-negative disease relapse. Similar findings were reported in a multicenter pediatric study utilizing three CAR T constructs (41BB, Kymriah and CD28z). Of 420 patients at a median of 12.4 years (range: 7–17.1 years), 17.9% received blinatumomab at a median of 129 days (range: 79–304 days) prior to CAR T-cell infusion. Patients who received blinatumomab were more likely to have CD19-dim antigen expression pre-CAR T therapy and were more likely to have residual disease post-CAR T therapy (18.3% vs. 7%). Median RFS and EFS in patients previously treated with blinatumomab was 20.3 months and 5.8 months vs. 44.9 months and 22.6 months, respectively, in the cohort who did not receive blinatumomab [79]. Considering the use of blinatumomab prior to CAR T-cell therapy demonstrates that patients may be at higher risk of CD19-negative disease, further investigation is required in the appropriateness of CAR T-cell therapy in those who received blinatumomab as part of their upfront chemotherapy treatment.

Preconditioning regimens also appear to play a pivotal role in the efficacy of CAR T-cell therapy. Generally, the role of such regimens is to deplete T cells following collection and create a microenvironment that is conducive to T-cell proliferation and stimulation upon infusion. Adequate lymphodepletion has been associated with a rise in serum monocyte chemoattractant protein (MCP-1), and patients with a greater increase in MCP-1 levels from baseline, have shown better PFS and CR rates [80].

Further, higher levels of IL-15 immediately following lymphodepletion have also been associated with better outcomes [81]. Further studies may elucidate optimal lymphodepletion strategies to facilitate the best microenvironment for CAR T-cell infusion.

Another possibly impactful pretreatment variable is high tumor burden (>15% CD19+ cells in the bone marrow). In one study, this was reported to improve B-cell aplasia and CAR durability when treated with CD19-41BB CAR; however, this finding was not reproducible [10]. In a meta-analysis of anti-CD19 CAR T-cell therapy in pediatric and adult patients with ALL, bone marrow involvement, disease status at the time of CAR T-cell infusion and presence of Philadelphia chromosome were not significantly associated with treatment outcomes [36]. Intrinsic patient factors may also affect the engraftment and proliferation of CAR T cells with one combined pediatric and adult study reporting higher levels of regulatory T-cells (Tregs) and the presence of non-CNS EM disease as independent risk factors for lower OS and RFS post-anti-CD19 CAR T therapy [15].

The immunosuppressive tumor microenvironment may also contribute to the early dysfunction, decreased expansion and poor persistence of CAR T cells. In preclinical studies, fourth generation CAR T-cells armored with IL-18 or CD40 ligand (CD40L) stimulated antitumor activity and downregulated exhaustion markers such as PD-1 in vitro [82,83].

The concurrent use of interleukin-12 (IL-12), a potent inflammatory cytokine produced by dendritic cells, macrophages and neutrophils is also being investigated as a potential strategy for ALL as well as other malignancies [84,85]. IL-12 results in the increased production of IFN γ, TNF α and enhances cytotoxic abilities of CD8^+^ T cells and natural killer (NK) cells to lyse tumor targets. Pegram et al. [86] demonstrated that umbilical cord blood (UCB) anti-CD19-specific CAR T cells with a 1928z/IL-12 construct led to enhanced proliferation and antitumor efficacy in mice, making them a promising strategy to enhance the durability of CAR T-cell therapy. Moreover, other cytokines such as IL-1, IL-23, and IL-36 have demonstrated potent anti-tumor capability indicating that the integration of these receptors into a CAR construct or the use of engineered IL compounds may have utility in developing a CAR T-cell product with increased cytotoxic ability [87]. For instance, when determining the use of engineered IL compounds, IL-2, a small compound which is generally secreted by CD4+ T cells, but also other T cells to a certain extent, has been shown to have utility in upregulating T-cell proliferation and effector state based on its interaction with the JAK-STAT pathway [88,89]. Indeed, engineering of compounds such as IL-2 and IL-15 are underway in order to influence CAR T cells in not only inhibiting CAR T-cell exhaustion but also increasing proliferation, persistence and cytotoxic ability through various intracellular transduction pathways [90,91]. Keeping in mind that while engineered IL compounds have utility in reversing or inhibiting T-cell exhaustion, the toxicity of these compounds must be appreciated. For instance, systemic use of IL-12 has been found to be associated with flu-like symptoms as well as severe bone marrow suppression and hepatic toxicity. However, researchers have demonstrated that through dose de-escalation and modification of dose scheduling, such toxicities can be mitigated [92].

## 7. Toxicities, Treatment and Prevention

CAR T therapy is associated with unique and potentially life-threatening toxicities, most notably CRS and ICANS. While early definitions for toxicities varied, in 2019, the American Society for Transplantation and Cellular Therapy (ASTCT) developed consensus criteria for the diagnosis and grading of CRS and ICANS in pediatric and adult patients that have been widely adopted [93].

CRS is an acute systemic inflammatory response due to supraphysiological cytokine elevation resulting in fever, hypoxia and hypotension. The reported incidence of CRS of any grade was 77% in patients receiving anti-CD19 or anti-CD22-directed CAR T therapy but has shown to vary across studies (Table 2) [18,21]. High vigilance for CRS is needed to allow early initiation of supportive care measures including fluid resuscitation, vasopressors, oxygen supplementation, cytokine blockade such as anti-IL-6 therapy and/or steroids when indicated. Patients should be rigorously monitored for progression of CRS. As CRS is currently a diagnosis of exclusion, other differential diagnoses must also be considered such as sepsis or adrenal insufficiency in order to institute appropriate management [94,95].

ICANS refers to the diverse group of neuro-toxic adverse effects associated with CAR T-cell infusion. They can range from minor symptoms such as subtle confusion to more concerning altered mental status, seizures and cerebral edema, occurring in 40–45% of patients receiving CAR T therapy (Table 2) [21,96]. While ICANS can occur concurrently with CRS or in isolation, the presence of CRS has been shown to be a strong predictor for ICANS [97,98]. The Cornell Assessment for Pediatric Delirium (CAPD) is a delirium screening tool that has been since incorporated in the ASTCT grading criteria and changes in the baseline CAPD score may be the earliest indicator of ICANS, though larger scale studies are needed [99]. ICANS-like CRS is a diagnosis of exclusion with no pathognomonic clinical or neuroimaging finding. Other causes of encephalopathy should be ruled out and management includes non-pharmacological methods to control of agitation, seizure prophylaxis, tocilizumab if occurring concurrently with CRS and corticosteroids. In both CRS and ICANS, other anti-cytokine therapy such as anakinra or activation of safety switches may be considered [94,95].

In a meta-analysis of pediatric and adult studies, the reported incidence of CRS or ICANS did not differ across anti-CD19 CAR T-cell constructs; however, it is difficult to directly compare the studies due to variations in the criteria used to grade CRS and ICANS [36]. Multiple factors have been associated with these toxicities including disease burden, degree of antigen expression, the affinity of the antigen-binding domain of the CAR T cell and the costimulatory domain. Modification of the CAR T cell is a potential strategy to decreasing toxicity. In a phase I trial (NCT02842138), Ying et al. [100] reported the use of an anti-CD19 CAR (CD19-BBz(86)) with a modified CD8α-derived hinge and transmembrane domains in 25 adult patients with refractory B-cell lymphoma with no patient experiencing greater than grade 1 CRS or ICANS. Other potential strategies being investigated include decreasing immunogenicity through the use of a fully humanized CAR T construct or via multi-targeted CAR T cells [69,101].

Although usually reversible, CRS and ICANS can both result in significant morbidity, with up to 47% of patients requiring intensive care unit admission [21]. Care teams should be properly trained to recognize patients with CRS and/or ICANS and clear lines of communication between the primary and the ICU team should be maintained to facilitate transfer of care if needed [102]. For the reasons stated above, investigation into CAR constructs with varying domains have utility in decreasing the rate of CRS/ICANS, and decreasing the incidence of ICU admission, as well as morbidity and mortality associated with CAR T therapy itself.

Chronic B-cell aplasia (BCA) is an on-target effect of anti-CD19 CAR T-cell therapy and should be monitored over time, especially in environments in which the anti-tumor effect of CAR T therapy is persistent. Indeed, based on a wide range of pediatric studies, the BCA can last more than 6 months in patients who demonstrate continued CR, and in certain cases, BCA persisted for as long as 4 years in some patients [103]. The resulting hypogammaglobulinemia predisposes a patient to infection; however, this can be managed with the use of chronic immunoglobulin infusion until the effect of the CAR T-cells wane over time [104]. More severely, progressive multifocal leukoencephalopathy (PML) due to John Cunningham virus (JCV) activation is a possible adverse effect due to chronic BCA resulting from anti-CD19 CAR T-cell therapy [105,106]. In fact, as CAR T-cell therapy becomes a more increasingly used treatment modality, cases of PML in patients following CAR T-cell therapy may be noted, as observed in the patient with R/R DLBCL who received anti-CD19 CART therapy [107].

Other reported adverse events include infusion reactions and infection (typically within the first 28 days) post-CAR T-cell infusion [108]. Late toxicities (>30 days post-infusion) include hypogammaglobinemia, prolonged cytopenias, secondary hemophagocytic lymphohistiocytosis, and the theoretical potential for insertional oncogenesis [95]. Allogeneic CAR T products also carry the risk of GVHD, as described prior. Longer term studies are needed to further understand the late physiological and psychological effects of CAR T therapy and modifications in the construct may help in relieving these issues.

Other mechanisms that have been studied in order to prevent CAR T cells from initiating a cascade of toxicities due to persistence is the use of small molecule switches. Researchers have demonstrated various ways in which CAR T cells can be engineered to possess receptors or epitopes that bind specific molecules, leading to turning off the effector function of the cell [109]. Conversely, through the use of peptide neo-epitopes (PNE), switchable CAR T cells (sCAR T) rely on the binding of a specific antibody for activation. A study showed the feasibility of this concept as the binding of a specifically engineered antibody to the PNE integrated onto the sCAR T surface led to the upregulation of cytokine release, such as IL-2, as well as other markers of T-cell activation such as CD25. However, this only occurred in the presence of CD19+ cells, indicating that the CAR T cell required both the target antigen and the switch in order to activate its effector function. This phenomenon was not observed in non-CAR T cells [110]. However, researchers have also demonstrated dual activity, in which dual receptors can be added to CAR T cells to either activate or inhibit function following stimulation of the respective receptor. This has been demonstrated in a study in which researchers developed a system in which CAR T cells were activated using rimiducid-inducible activation of MYD88 and CD40 signaling. Conversely, CAR T cells were inhibited through rapamycin-induced apoptosis of the T cell in order to downregulate toxicity from therapy [111]. These studies indicate that the development of a CAR T-cell switch has vast utility in developing T cells that are susceptible to small molecule activation as well as inhibition in order to mitigate the effects of CAR T-cell-induced toxicity.

## 8. Predictive Biomarkers for Toxicities and Disease Relapse

Extensive application of CAR T therapies may be limited by unique toxicities and treatment-related mortality. Identifying biomarkers that may aid in the prediction of these toxicities are urgently needed to optimize patient care. Teachey et al. [112] evaluated serial potential biomarkers from 39 children and 12 adults with R/R ALL post-anti-CD19-directed CAR T therapy. They proposed several combinations of biomarkers including IFNγ, sgp130, and sIL1RA. IFNγ, IL13, and MIP1α, sgp130, MCP1, and Eotaxin, demonstrated varying degree of sensitivity (86–100%) and specificity (89–97%) in predicting grade 4–5 CRS. However, differentiating CRS from infection can be a diagnostic challenge. In this setting, elevated levels of IFNγ in isolation or in combination with other biomarkers such as IL6, sIL2Rα or IL1β may be helpful in clinical practice to help differentiate CRS from sepsis. IFNγ is not expected to be elevated in the setting of infection [112,113]. Luo et al. [114] also reported “double peaks of IL-6” as a specific sign of grade 4–5 infection and the combination of IL-8, IL-1β and interferon-γ as a predictive model for life-threatening infection with a sensitivity of 100% and specificity of 82.8%. Higher cytokine values of TNF-α, IL-6, IL-8 and IL-15 have also been reported to be associated with worse neurotoxicity [96]. Other potential biomarkers reported in the development of ICANS include ApoA1 and angiogenin [115].

Understanding risk factors for developing toxicities post-CAR T-cell therapy is also imperative as disease burden alone does not predict the likelihood of developing CRS [112]. Additionally, disease relapse is a major cause of morbidity and mortality post-CAR T-cell therapy and identification of risk factors for disease relapse is urgently needed [72]. Despite 81% of patients achieving MRD negativity, up to 50% of these patients relapsed at 12 months post-infusion in the ELIANA trial [21]. Of note, there are multiple methods for detecting MRD with varying sensitivity of 10^−3^ to 10^−6^ and therefore the method used for detection is important. Gardener et al. [10] reported that in 40 patients who achieved MRD negativity by multiparameter flow cytometry, 27 of them had a malignant clone identified by next generation sequencing (NGS). Further studies are needed to improve the detection of submicroscopic disease and also identify predictive biomarkers and models both for disease relapse and toxicities to potentially guide early intervention and develop preventative strategies. Future studies may investigate the utility of an algorithmic stratification of NGS MRD detection and early loss of BCA to determine which patients may benefit from post-CAR T therapy consolidation strategies such as HSCT. Preliminary studies indicate NGS of MRD in patients following CAR T-cell therapy has great utility, regardless of BCA status. This includes patients that relapse with BCA but CD19-negative disease. In these cases, NGS on a bone marrow sample to determine MRD status remains practical as the sequencing detected in tumor cells by NGS is not affected [116].

## 9. Financial Cost of CAR T-Cell Therapy

CAR T therapy carries a significant financial burden along with uncertainty of its long-term benefits. The manufacturing cost of tisagenlecleucel alone, excluding pre-infusion screening, apheresis, hospitalization and supportive measures as required for routine care or for CAR T-cell therapy-related complications is $475,000 USD [117]. While its short-term therapeutic value is widely recognized, the economic burden remains an area of concern. Nonetheless, several studies have concluded that tisagenlecleucel can be a cost-effective treatment option per quality of life years gained from both a payer and a societal perspective [118,119,120,121,122]. Additionally, despite its high upfront cost, overall resource utilization in patients receiving CAR T-cell therapy may be similar to patients with ALL receiving other standard therapies [123,124]. Long-term studies, however, are needed to truly understand outcomes and validate the cost-effectiveness of CAR T therapy.

## 10. Conclusions and Future Directions

**Figure 2:**
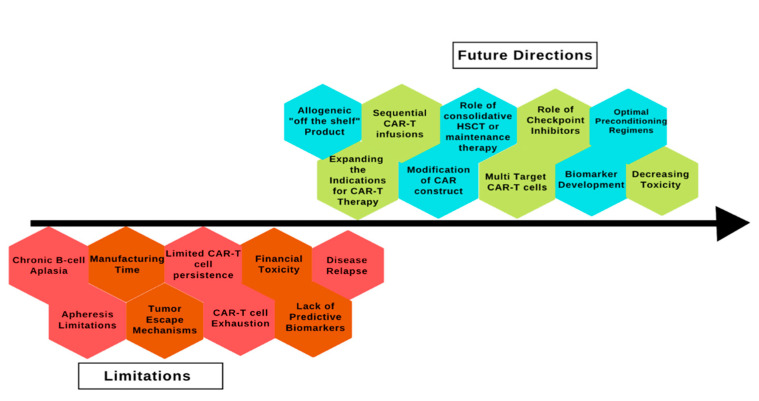
Limitations and future directions of chimeric antigen receptor therapy in acute lymphoblastic leukemia. CAR: chimeric antigen receptor; HSCT: hematopoietic stem cell transplant.

CAR T-cell therapy has revolutionized the treatment of ALL but despite being a promising treatment option, several limitations must be overcome to increase its utility and access (Figure 2).

These include manufacturing challenges such as weight limitations; particularly in pediatric patients, unsuccessful apheresis in heavily pretreated patients may lead to insufficient lymphocytes to manufacture an effective product and unstable disease where waiting for the genetically modified cells is not an option. In the ELIANA trial, 7.6% of patients failed to receive the CAR T-cell product due to “product related issues” and 7.6% died prior to receiving the CAR T cells [21]. However, as an alternative, Rossoff et al. [125] reported similar efficacy in 24 patients who received tisagenlecleucel out of product specifications compared to those who received standard-of-care products (OS at 12 months: 85% vs. 70%, respectively, and EFS at 12 months of 55 vs. 51%, respectively). While larger- scale studies are needed to define product specifications, off-the-shelf products are a more readily available alternative to manufactured autologous products. Ideally, these products must evade host-mediated destruction as well as avoid the risk of GVHD. Allogeneic products such as UCAR-T19, UCAR-T22 and genetically modified natural killer (NK) cells are promising alternatives that may not only provide access to patients when apheresis is not feasible but also remove the potential concern for infusion of a product with contamination with tumor cells [126,127,128].

Other strategies currently being explored focus on improving CAR T-cell persistence, enhancing engraftment durability and evading current tumor escape mechanisms. PLAT-03 is an actively recruiting clinical trial evaluating the effect of administering autologous T-cell antigen-presenting cells (T-APCs) following CAR T-cell infusion on anti-CD19 CAR T-cell persistence and disease relapse [129]. Dual and triple target CAR constructs are also being investigated to combat CD19-negative disease relapse as previously mentioned. Researchers are also currently exploring the use of molecular switches that act to mitigate or stimulate CAR T-cell function when needed.

Given its highly promising results, several pediatric ALL protocols are evaluating the role of CAR T therapy as a frontline treatment option. The Children’s Oncology Group is conducting a phase II trial of tisagenlecleucel as first line therapy in patients ≤25 years with B-cell ALL who are MRD-positive at the end of consolidation therapy (AALL1721). The St. Jude TOT17 protocol uses 19-BBz CAR T cells for those patients with B-ALL and MRD of 1% or more at the end of induction or isolated CNS relapse [6,130].

Future research, however, is needed to improve the efficacy, mitigate toxicity, define the role of consolidative HSCT post-CAR T therapy and reduce cost while improving access to care for this highly revolutionary therapeutic option for patients with ALL.

## Figures and Tables

**Table 1:** Generations of chimeric antigen receptor T cells for acute lymphoblastic leukemia.

Generation	Intracellular Costimulatory Signaling Domains
1st	CD3 ζ
2nd	CD3 ζ and one costimulatory domain (CD28 or CD27 or 4-1BB or OX40(CD134))
3rd	CD3 ζ and two costimulatory domains
4th	2nd generation CAR T-cell and cytokine/costimulatory ligand transgene with IL12 expression
5th	Similar to 4th generation CAR construct with addition of intracellular domain of the IL-2 receptor

CD: cluster of differentiation; ζ: zeta; CAR T: chimeric antigen receptor T cell.

**Table 2:** Summary of clinical trials of chimeric antigen receptor T-cell therapy for acute lymphoblastic leukemia in the pediatric and AYA population [10,15,16,17,18,19,20,21,22,23,24,25,26,27,28,29,30,31,32].

Chimeric Antigen Receptor T-Cell Therapy Trials for Acute Lymphoblastic Leukemia in the Pediatric and AYA Population							
Author Year Trial NCT#	CAR T Construct (Target Antigen/Costimulatory Domain)	Study Population (No of Patients & Median Age)	Study Design	Clinical Response	Duration of CAR T Persistence	HSCT Post CAR T Therapy	CRS and ICANS (Grade ≥3)
Myers et al. 2021HuCAR T19NCT02374333	CD22/ 4-1BB/humanized anti-CD19 scFv domain	n = 74 (B cell ALL, n = 72, B-LLy, n = 2) CAR Naïve cohort, n = 41 10.3 y (1.7–29.1 y) Retreatment cohort, n = 33 12.6 y(4.4–24.8 y)	Phase 1	CAR Naïve Cohort: At Day 28: CR: 98% At 12 months: RFS 84% OS:90% At 24 months: RFS: 74% OS: 88% Retreatment cohort: At Day 28: CR: 79% At 12 months: RFS: 74% OS:76% At 24 months: RFS: 58% OS: 55%	6-month cumulative incidence of loss of CAR T persistence:CAR naïve cohort: 27% Retreatment cohort 48%	CAR Naïve Cohort: 4 patients proceeded to HSCT Retreatment Cohort: 1 patient proceeded to HSCT	CAR Naïve Cohort: CRS: 14.6% ICANS: 7.3% Retreatment cohort: CRS: 15.2% ICANS: 0%
Shah et al. 2021 NCT01593696	CD-19/ CD28	n = 50 13.5 y (4.3–30.4)	Phase 1	Median follow-up at 4.8 years OS: 10.5 months	NA	21 of 28 patients who achieved MRD negative disease proceeded to HSCT. 2 patients subsequently had DR All 7 patients who did not receive HSCT had DR	CRS: 18% ICANS: 4%
Pasquini et al. 2020	CD-19/ 4-1BB	n = 255 13.2 y (0.4–26.1 y)	Prospective Noninterventional	At 12 months: EFS: 52.4% ^1^ OS: 77.2%	NA	34 patients proceeded to HSCT	CRS 16.1% ICANS: 9%
Ma et al. 2019 NCT02963038	CD-19/ CD28 and 4-1BB	n = 10 6.5 y (3–13 y)	Phase 1/2	At 12 months: OS: 40%	NA	NA	CRS: 40% ICANS:30%
Ghorashian et al. 2019 CARPALL Trial NCT02443831	Lower affinity CD19 CAR (CAT)/ 4-1BB	n = 14 9.24 y (1.4 to 19.3 y)	Phase 1	At 12 months: EFS: 46% ^2^ OS: 63%	215 days	NA	CRS: 0% ICANS: 0%
Curran et al. 2019 NCT01860937	CD-19/ CD28	n = 25 13.5 y (1–22.5 y)	Phase 1	NA	NA	15 proceeded to HSCT, 2 had DR (17.5 and 27.5 months after CAR T therapy)	CRS: 16% ICANS: 28%
Fry et al. 2018 NCT02315612	CD-22/ 4-1BB	n = 21 19 y (3–30 y)	Phase 1	At 12 months: CR: 73% of patients	NA	NA	CRS: 0% ICANS: 0%
Gardner et al. 2017 PLAT-02 Trial NCT 02028455	CD-19/ 4-1BB Patients receive 1:1 ratio of CD4:CD8 CAR T cells	n = 45 12.3 y (1.3–24.4 y)	Phase 1/2	At 12 months: RFS: 50.8% OS: 69.5%	6.4 months (measured by B cell aplasia as a surrogate marker of T cell persistence)	11 proceeded to HSCT and 2 had disease recurrence	CRS 23% ICANS: 21%
Shah et al. 2016	CD-22/ 4-1BB	n = 9 20 y (7–22 y)	Phase I	At Day 28 CR: 44%	NA	NA	CRS: 0% ICANS: 0%
Maude et al. 2014 ELIANA Trial NCT02435849	CD-19/ 4-1BB	n = 75 11 y (3–23 y)	Phase 2	At 12 months: RFS: 59% OS: 76%	168 days (20–617 days)	8 patients proceeded to HSCT	CRS: 46% ICANS: 13%
Combined Adult and Pediatric Clinical Trials of CAR T-Cell Therapy for Acute Lymphoblastic Leukemia							
Shah et al. 2021 ZUMA-3 study NCT02614066	CD-19/ CD28	n = 45 46 y (18–77 y)	Phase 1	Median OS 12.1 months Median RFS 7.3 months	NA	NA	CRS 31% ICANS 38%
Singh et al. 2021 NCT02588456 NCT02650414	CD-22/ 4-1BB	n= 9 (6 children, 3 adults) Age not specified	Phase 1	At 12 months: CR: 50%	NA	NA	CRS: 12.5% ICANS:0%
Heng et al. 2020 NCT02349698	CD19/ CD137 (4-1BB)	n = 10 18.8 y (5–40 y)	Phase 1/2	At 6 months: OS: 100% Leukemia-free survival: 90%	NA	2 patients proceeded to HSCT	CRS 40% ICANS 40%
Frey et al. 2020 NCT02030847 NCT01029366	CD-19/ 4-1BB	n = 35 33.8 y (20.6–70.4 y)	Phase 1/2	At a median follow-up of 13 months: Median OS: 19.1 months Median EFS: 5.6 months	NA	9 patients proceeded to HSCT	CRS 72% ICANS 6%
An et al. 2020 NCT02735291	CD-19/ CD137 (4-1BB)	n = 47 22 y (3–72 y)	Phase 2	At 12 months: OS: 53% RFS: 45%	85 days	10 patients proceeded to HSCT	CRS 23.4% ICANS 2.1%
Roddie et al. 2020 NCT02935257	CD19/ 4-1BBz	n = 19 43 y (18–62 y)	Phase 1	At a median follow-up of 12.2 months: DFS: 58%	NA	2 patients proceeded to HSCT	CRS: 0% ICANS: 16%
Park et al. 2018 NCT01044069	CD-19/ CD28	n = 53 44 y (23–74 y)	Phase 1	At a median follow-up of 29 months: Median OS 12.9 months Median EFS ^3^ 6.1 months	14 days	17 patients proceeded to HSCT (5 alive, 6 had relapsed disease, 6 died from transplant related toxicities)	CRS 26% ICANS 42%
Turtle et al. 2016 NCT01865617	CD-19/ CD28 & 4-1BB 1:1 ratio of CD4:CD8 CAR T cells	n = 32 40 y (20–73 y)	Phase 1/2	NA	NA	13 patients proceeded to HSCT	CRS: 23% ICANS: 50%
Chang et al. 2016	CD28/CD137/CD27/CD3ζ-iCasp9 (4SCAR-19) Auto and allo CAR	n = 102 Pediatric: n= 55; 9 y (2–17 y) Adult; n = 47; 37 y (19–70 y) Cohort 1: Blasts < 50%; n = 69 Cohort 2: Blasts > 50%; n = 33	Phase 1/2	At median follow-up of 7 months: CRCohort 1: 91.3% Cohort 2: 75.8%	NA	NA	CRS: 10.7%

^1^ Time from tisagenlecleucel infusion to death resulting from any cause, relapse, or treatment failure (failure to achieve remission, including death without remission), whichever occurred first. ^2^ Time from CAR T cells to the following events: no response or morphological relapse after having CR or CR with incomplete hematologic recovery. ^3^ Defined as no response, relapse, or death as the event, whichever occurred first. AYA: adolescent and young adult; NCT: national clinical trial; CAR T: chimeric antigen receptor T cell; HSCT: hematopoietic stem cell transplant; CRS: cytokine release syndrome; ICANS: immune effector cell–associated neurotoxicity syndrome; CD: cluster of differentiation; ALL: acute lymphoblastic leukemia LLy: lymphoblastic lymphoma; y: years; CR: complete remission; OS: overall survival; RFS: relapse-free survival; MRD: minimal residual disease; DR: disease relapse; EFS: event-free survival; DFS: disease-free survival.

**Table 3:** Summary of clinical trials of bispecific chimeric antigen receptor T-cell therapy for acute lymphoblastic leukemia [70,71,72,73,74].

Trial	CAR T Construct (Target Antigen/Costimulatory Domain)	Study Population (No of Patients & Median Age)	Study Design	Clinical Response	Duration of CAR T Persistence	CRS and ICANS (Grade ≥ 3)
Cordoba et al. 2021 AUTO3 AMELIA trial NCT03289455	CD-19 & CD-22/ OX-40 & 4-1BB	n = 15 8.y (4–16 y)	Phase 1/2	CR at 1 month: 86% At 12 months: OS: 60% EFS: 32%	Median time to last detection in blood: 119 days	CRS: 0% ICANS: 0%
Dai et al. 2020 NCT03185494	CD-19 & CD-22/ 4-1BB	n = 6 27.8 y (17–44 y)	Phase 1	100% CR at day 30	NA	CRS: 0% ICANS: 0%
Schultz et al. 2019 NCT03233854 NCT03241940	CD-19 & CD-22/ 4-1BB	n = 19 23 y (2–68 y)	Phase 1	OS: 92% at 9.5 months	NA	CRS: 8% ICANS: 8%
Yang et al. 2019 NCT03825731	CD-19 & CD-22/ 4-1BB	n = 16 1–45 y	Phase 1	100% CR at day 15 for patients in the medium dose group	NA	CRS: 0% ICANS: 0%
Gardner et al. 2018 NCT03330691	CD-19 & CD-22/ 4-1BB	n = 7 1–26 y	Phase 1	CR in 71% of subjects at 12 months	NA	CRS: 0% ICANS: 0%

CAR T: chimeric antigen receptor T cell; CRS: cytokine release syndrome; ICANS: immune effector cell–associated neurotoxicity syndrome; CD: cluster of differentiation; y: years; CR: complete remission; OS: overall survival; EFS: event-free survival.
